# The Synthetic Opioid Fentanyl Increases HIV Replication and Chemokine Co-Receptor Expression in Lymphocyte Cell Lines

**DOI:** 10.3390/v15041027

**Published:** 2023-04-21

**Authors:** Janani Madhuravasal Krishnan, Ling Kong, Rebekah Karns, Mario Medvedovic, Kenneth E. Sherman, Jason T. Blackard

**Affiliations:** 1 Division of Digestive Diseases, Department of Internal Medicine, University of Cincinnati College of Medicine, Cincinnati, OH 45267, USA; jananimk@ucmail.uc.edu (J.M.K.);; 2 Digestive Health Center, Cincinnati Children’s Hospital, Cincinnati, OH 45229, USA; 3 Department of Environmental & Public Health Sciences, University of Cincinnati College of Medicine, Cincinnati, OH 45267, USA; 4 Center for Addiction Research, University of Cincinnati College of Medicine, Cincinnati, OH 45267, USA

**Keywords:** opioid, fentanyl, drug use, HIV

## Abstract

Background: In the United States, the illicit use of synthetic opioids such as fentanyl has led to a serious public health crisis. Synthetic opioids are known to enhance viral replication and to suppress immunologic responses, but their effects on HIV pathogenesis remain unclear. Thus, we examined the impact of fentanyl on HIV-susceptible and HIV-infected cell types. Methods: TZM-bl and HIV-infected lymphocyte cells were incubated with fentanyl at varying concentrations. Expression levels of the CXCR4 and CCR5 chemokine receptors and HIV p24 antigen were quantified with ELISA. HIV proviral DNA was quantified using SYBR RT-PCR. Cell viability was detected with the MTT assay. RNAseq was performed to characterize cellular gene regulation in the presence of fentanyl. Results: Fentanyl enhanced expression of both chemokine receptor levels in a dose-dependent manner in HIV-susceptible and infected cell lines. Similarly, fentanyl induced viral expression in HIV-exposed TZM-bl cells and in HIV-infected lymphocyte cell lines. Multiple genes associated with apoptosis, antiviral/interferon response, chemokine signaling, and NFκB signaling were differentially regulated. Conclusions: Synthetic opioid fentanyl impacts HIV replication and chemokine co-receptor expression. Increased virus levels suggest that opioid use may increase the likelihood of transmission and accelerate disease progression.

## 1. Introduction

The number of opioid overdose deaths in the United States has increased dramatically in recent years [1,2,3]. Fentanyl or fentanyl analogs were involved in ~90% of unintended overdoses [3,4,5,6]. Opioids are endogenous, exogenous, or synthetic substances that work by activating multiple opioid receptors, and these opioid receptors are present on lymphocytes, monocytes, macrophages, and neutrophils, as well as other immune cells [7,8,9]. As opioids alter both innate and adaptive immune functions, it is critical to understand how opioid use affects distinct immune cell types [10,11,12]. Fentanyl is ~50 times more potent than heroin and ~100 times more potent than morphine [13]. Compared with heroin, fentanyl has a rapid onset but shorter duration of action, which increases the frequency with which people inject drugs and increases drug sharing. Injection drug use increases the risk of transmission of blood-borne viruses such as HIV, hepatitis C virus, and hepatitis B virus [13,14,15,16].

HIV enters target cells by forming a complex consisting of the viral envelope glycoprotein (gp120), CD4 receptor, and members of the chemokine co-receptor family (e.g., CCR5 and CXCR4). Multicellular immune responses (e.g., natural killer cells and T regulatory cells) are regulated by CCR5 [17]. Multiple pathological conditions are associated with CXCR4, including immune system disorders, viral infections, and cancer [18]. Amphetamines, cocaine, marijuana, and opiates are all cofactors that increase the risk of HIV infection and disease progression [19,20]. For instance, morphine enhances HIV replication in vitro [21,22,23,24,25] by upregulation of the chemokine co-receptor CCR5 [26]. Morphine downregulates the level of beta chemokines, regulates the expression of pro-inflammatory markers such as TNF-α and IL-6 to block CD8-mediated anti-HIV activity, inhibits the expression of anti-HIV miRNA, and activates the HIV long terminal repeat (LTR) [27,28,29,30]. Non-opioid drugs of abuse also impact HIV replication and disease progression. For example, cocaine promotes HIV infection and replication by suppressing protective chemokines and/or upregulating HIV co-receptors [31]. Methamphetamine also enhances HIV replication by suppression of immune cells in vivo [32].

Although fentanyl, fentanyl analogs, and fentanyl metabolites are commonly detected in persons experiencing overdoses, their effects on viral replication are poorly characterized. The proliferation of viruses can be enhanced by several drugs of abuse that suppress immunological responses; the effects of fentanyl on replication of HIV and cellular gene expression have not been investigated thoroughly. Thus, we evaluated the effects of fentanyl on HIV-susceptible/infected cell types and chemokine receptor expression. As CD4^+^ T cells represent a major reservoir of HIV replication, we also assessed whether fentanyl affected HIV replication in CD4^+^ T cells.

## 2. Methods

### 2.1. Cell Lines and Reagents

TZM-bl is an indicator cell line that expresses high levels of CD4, CCR5, and CXCR4 and is susceptible to infection with a variety of HIV isolates. TZM-bl cells incorporate two HIV Tat-regulated reporter genes—firefly luciferase and *b*-galactosidase—that facilitate quantification of infectious HIV [33,34]. J-Lat GFP is a Jurkat-derived T lymphocyte cell line with a single HIV retroviral vector insertion site that enables LTR-driven green fluorescent protein (GFP) expression [35,36]. H9 (cat.no. ARP-400) is derived from the human T cell line HUT 78 and is infected HIV-1 IIIB. ACH-2 is a T lymphocyte cell line containing integrated LAV provirus [37,38]. All cell lines were provided by the NIH AIDS Reagent Program. TZM-bl cells were cultured in Dulbecco’s modified Eagle medium (DMEM) with 10% fetal bovine serum (FBS), 100 U/mL penicillin, 100 μg/mL streptomycin, and 1% 200 mM L-glutamine at 37 °C and 5% CO_2_. T cell lines were maintained in RPMI 1640 medium (Life Technologies, Grand Island, NY, USA) supplemented with 10% heat-inactivated FBS, 100 U/mL penicillin, and 100 μg/mL streptomycin (Life Technologies) at 37 °C and 5% CO_2_.

As a certified reference material from Cerilliant (Round Rock, TX, USA), fentanyl can be used for the testing, calibration, and quantification in analytical and research applications [39]. The Institutional Review Board at the University of Cincinnati approved the use of human blood as part of protocol 0584_2019 (approved 29 May 2019).

### 2.2. Quantification of Mu Opioid Receptor

Mu opioid receptor (MOR) expression was quantified in SH-5YSY, TZM-bl, J-Lat GFP, ACH-2, and H9 cells. Cell lysates were prepared from ~1 × 10^5^ cells, and 100 uL of the lysate was processed for human opioid receptor mu 1 (OPRM1) ELISA (My BioSource; San Diego, CA, USA).

### 2.3. Propagation of HIV

Infectious HIV_YK-JRCSF_ and HIV_NL4-3_ were prepared by transfection of 1 × 10^6^ 293T cells (ATCC #CRL-3216) per well with 2 μg of the full-length HIV_YK-JRCSF_ and HIV_NL4-3_ plasmids, obtained from the NIH AIDS Reagent Program, with the FuGene6 transfection reagent (Roche; Basel, Switzerland). Transfected cells were incubated at 37 °C for 48 h. The supernatant was harvested and passed through a 0.20 μm filter to remove cellular debris and then precipitated in polyethylene glycol at 4 °C. Precipitated virus was centrifuged at 3000 g for 20 min, and the virus precipitate was resuspended in phosphate-buffered saline (PBS) and preserved at −80 °C. The virus was titered using TZM-bl cells and β -galactosidase staining. HIV p24 protein in cell culture supernatants was quantified as outlined below.

### 2.4. HIV Infection, Drug Exposure, and p24 Protein Quantification

TZM-bl cells were seeded at ~2 × 10^5^ cells per well. After 24 h, cells were treated with HIV_YK-JRCSF_ and HIV_NL4-3_ at TCID_50_ of 0.5 for 1 h. Prior to the exposure to fentanyl, the cells were rinsed with PBS three times to remove unbound virus and -replaced with fresh media. Working concentrations of fentanyl were prepared by dilution of the concentration stock with dH_2_O to obtain 1 ng/mL, 100 ng/mL, and 10 ug/mL concentrations, and added to the respective wells. After incubation with the drug for 24 h, the expression of HIV p24 protein levels was quantified in cell culture supernatants using the HIV p24 ELISA Kit (Abcam; Cambridge, MA) with a lower limit of sensitivity of 1.1 pg/mL.

### 2.5. Quantification of Integrated HIV DNA

TZM-bl cells were seeded at 1 × 10^6^ cells per well. After 24 h, the cells were treated with HIV_NL4-3_ at TCID_50_ of 0.5 for 1 h, rinsed with PBS three times to remove unbound virus, and - replaced with fresh media. Fentanyl at varying concentrations was added to the respective wells and incubated. After incubation with the drug for 24 h, cellular DNA was extracted from cells using the Qiagen mini kit per the manufacturer’s instructions. DNA was similarly extracted from ACH-2 cells that contain a single copy of HIV-1 proviral DNA (LAV strain) [37,38] and used as a positive control to quantify virus levels. The number of HIV-1 proviral copies was quantified with real-time PCR amplification using Brilliant III ultra-fast SYBR green QPCR master mix (Agilent), as described elsewhere [40]. Real-time PCR was performed using SYBR green 2× Master Mix with 200 nM of each oligonucleotide primer targeting the HIV-1 pol gene and DNA extracted from cells treated and untreated with HIV_NL4-3_ +/− fentanyl at concentrations of 1 ng/mL, 100 ng/mL, and 10 ug/mL. To quantify HIV-1 provirus, a standard curve was defined, using serial dilutions of ACH-2-derived DNA ranging from 1 to 10^6^ copies per cell. All standard dilutions, controls, and samples were run in duplicate, and the average value ct was utilized to quantify HIV-1 DNA copies.

### 2.6. Cell Viability

TZM-bl, J-Lat GFP, ACH-2, and H9 cells were seeded at a concentration of 5 × 10^4^ cells per well in 100 μL of DMEM medium + 10% FBS and allowed to adhere for 24 h at 37 °C. After 24 h of incubation, fentanyl was added. At 24 h post fentanyl exposure, cell viability was evaluated with a 3-(4,5-dimethylthiazol-2-yl)-2,5-diphenyl tetrazolium bromide (MTT) assay using the MTT Cell Proliferation Assay Kit (Abcam; Cambridge, MA, USA).

### 2.7. Chemokine Receptor Expression

A quantity of ~2 × 10^5^ TZM-bl, J-Lat GFP, ACH-2, or H9 cells were seeded, and after 24 h fentanyl was added. Post fentanyl exposure, cells were harvested and freeze-thawed three times to prepare cell lysates. CXCR4 or CCR5 receptor protein (pg/mL) was quantified in cell lysates with ELISA (My BioSource; San Diego, CA, USA).

### 2.8. Primary Cell Isolation and Culture

Peripheral blood samples were collected from healthy adult donors with no self-reported HIV or HCV at the University of Cincinnati Medical Center. Patients provided written informed consent prior to any study procedures. Four mLs of blood was collected in BD Vacutainer Cell Preparation Tubes (catalog #362760). After inverting the tubes 8–10 times, the tubes were centrifuged for 25 min at 1900 RCF. Mononuclear cells were collected in 15 mL centrifuge tubes and rinsed with PBS two times. The final cell pellet was resuspended in 5 mLs of PBS, and the total number of cells in the suspension was determined by trypan blue staining.

Naïve CD4^+^ T cells were subsequently isolated by negative selection using the naïve CD4^+^ T cell isolation kit (cat. no. 130-096-533, Miltenyi Biotec GmbH, Bergisch Gladbach, Germany) according to the manufacturer’s instructions. Briefly, the PBMC cell suspension was centrifuged at 800× g for 5 min. The cell pellet was resuspended in 40 μL of MACS running buffer (PBS + 5%FBS) per 1 × 10^7^ cells. An amount of 10 μL of CD4^+^ T cell biotin-antibody cocktail was added per 1 × 10^7^ cells and incubated at 4 °C for 10 min. An amount of 30 uL of MACS running buffer and 20 uL of anti-biotin microbeads per 1 × 10^7^ cells were added and incubated at 4 °C for 10 min. Cells were resuspended in MACS running buffer and applied to an LS column placed in a magnet. Unlabeled CD+ T cells were collected and centrifuged at 800× g for 5 min. The cells were resuspended with 1 mL of MACS running buffer, and the total cell count was determined by trypan blue staining. Cells were centrifuged again and resuspended in 1 mL RPMI + 10% FBS + 1% antibiotics (Pen/Strep) + 1% glutamine. Purified cells were plated at 0.3 × 10^6^ cells per well in a 96-well plate with 200 ul of RPMI + 10% FBS + 1% antibiotics (Pen/Strep) + 1% glutamine. The cells were stimulated with anti-CD3/CD28 dynabeads (cat. no. 111.31D, Invitrogen, 1 cell per 3 beads) + 100 IU/mL IL−2 at 37 °C in 5% CO_2_ for 3 days.

After 3 days of incubation, the activated cells were collected and transferred to a 5 mL polystyrene tube and placed in a dynabead magnet to remove beads from cells. The cells were then transferred to a new tube and centrifuged at 800× *g* for 5 min. The cell pellet was resuspended in 1 mL of RPMI + 10% FBS + 1% antibiotics (Pen/Strep) + 1% glutamine, and the cell count was determined. An amount of ~1 × 10^5^ cells was seeded per well. Cells were infected with HIV_NL4-3_ at MOI of 1 for 2 h and rinsed with RPMI + 10% FBS + 1% antibiotics (Pen/Strep) + 1% glutamine three times to remove any unbound virus and - replaced with fresh media. Fentanyl at concentration of 10 ug/mL was added to the respective wells and incubated at 37 °C in 5% CO_2_. After incubation with the drug and virus for 72 h, HIV proviral DNA was quantified in cells with real-time PCR based on SYBR Green I detection, and HIV p24 antigen expression was estimated from the cell culture supernatant.

### 2.9. Cellular RNA Isolation and Purification

Total RNA from fentanyl-treated (10 μg/mL) and untreated ACH-2 cells was isolated using a commercially available miRNA Isolation Kit (miVana; Applied Biosystems; Carlsbad, CA, USA) according to the manufacturer’s protocol. A NanoDrop 2000 spectrophotometer (Thermo Fisher Scientific; Waltham, MA, USA) was used to determine total RNA concentration and purity. A 2100 Bioanalyzer (Agilent; Santa Clara, CA, USA) and agarose electrophoresis were used to assess RNA integrity.

### 2.10. miRNA-Seq and Data Analysis

MicroRNA-seq was performed by the Genomics, Epigenomics and Sequencing Core at the University of Cincinnati [41]. A NEBNext small RNA sample library preparation kit (NEB; Ipswich, MA, USA) was used to prepare the library with a modified approach for precise miRNA library size selection; as a result, the kit could process low-quality RNA with limited input for higher library recovery and miRNA read alignment. After 15 cycles of PCR with 100 ng total RNA as input, the libraries with unique indices were then pooled, column-cleaned, and combined with a customized DNA ladder containing 135 and 146 base pairs (bp) purified PCR amplicon. This size range corresponds to a 16–27 nt insert miRNA library that covers all miRNAs. Agarose gel electrophoresis and gel excision were performed. The library pool ranging, from 135 to 146 bp, including the DNA marker, was gel-purified and quantified using the NEBNext Library Quant kit (NEB) in the Quant Studio 5 Real-Time PCR System (Thermo Fisher Scientific; Waltham, MA, USA). The first round of sequencing was performed using an Illumina Nextseq 550 sequencer to generate a few million reads to measure the relative concentration of each library. For the final data analysis, the capacity of each library was adjusted to generate the predicted number of equal reads from each sample. Sequence reads were pre-processed to remove adapters and filter low-quality reads using the ShortRead R package [42]. Reads were aligned to the reference human genome (hg19) using the Bowtie2 aligner [43]. The reads aligning to each known mature miRNA were counted using Bioconductor packages for next-generation sequencing data analysis [44] based on miRNA definitions in the miRBase database [45]. Statistical analysis to detect differentially expressed miRNAs were performed, and *p*-values were calculated based on the negative binomial model of read counts as implemented in the edgeR [46] R package.

### 2.11. RNAseq Analysis

The Genomics, Epigenomics and Sequencing Core at the University of Cincinnati performed directed polyA RNA-seq, following established protocols [47,48]. Bioanalyzer was used to test the quality of total RNA (Agilent; Santa Clara, CA, USA). The Poly(A) mRNA Magnetic Isolation Module (NEBNext; Ipswich, MA, USA) was used to isolate polyA RNA for library preparation with 1 µg of high-quality total RNA as input. SMARTer Apollo automated NGS library prep system (Takara Bio USA; Mountain View, CA, USA) was used for enrichment of polyA RNA. For library preparation, the New England BioLabs NEBNext Ultra II Directional RNA Library Prep kit (PCR cycle number 8) was used. The individual indexed libraries were proportionally pooled and sequenced on the NextSeq 550 sequencer (Illumina; San Diego, CA) with the setting of single read 1 × 85 bp after QC and quantification via real-time qPCR (NEBNext Library Quant Kit; New England BioLabs; Ipswich, MA, USA).

Raw reads were processed using Kallisto, which employs pseudoalignment to quickly and accurately determine if reads are compatible with genomic targets. Genomic annotations were provided by the University of California, Santa Cruz (UCSC) Genome Browser, with output as transcripts per million (TPM). Raw data were log2-tranformed, the baseline was set as the median of all samples. Further filtering was performed to include only transcripts with TPM > 3 in 50% of samples (N = 10,703 transcripts). The level of differential expression was determined using moderated *t*-tests with a significance cutoff of *p* < 0.05. Significant transcripts were assessed for ontological significance using ToppGene and ToppCluster, with figures generated in Cytoscape, focusing on biological processes and pathways. To construct comprehensive gene sets for candidate biological functions, Gataca (https://gataca.cchmc.org/gataca/; accessed on 24 April 2022) and ToppGene were used (https://toppgene.cchmc.org; accessed on 24 April 2022), where the input for Gataca is a biological process or pathway and the output is related genes. Candidate gene sets were then used to generate principal components through principal component analysis (PCA), which were plotted to determine each candidate gene set’s ability to segregate samples according to drug exposure.

### 2.12. Statistical Analysis

The standard deviation of each experimental condition was represented by error bars for technical duplicates. An ANOVA with replication was used to evaluate the statistical significance (*p* < 0.05) of the different fentanyl doses compared to no drug. Statistix v10 (Analytical Software, Tallahassee, FL, USA) was used to perform all the statistical analysis.

## 3. Results

### 3.1. HIV-Susceptible and HIV-Infected Cell Lines Express Mu Opioid Receptor

**Figure 1:**
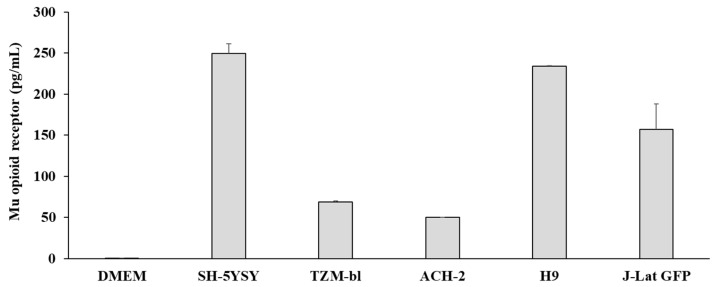
Expression of mu opioid receptor was quantified with ELISA in ~1 × 10^5^ cells. DMEM = Dulbecco’s Modified Eagle Medium (DMEM). Error bars denote standard deviation of three independent experiments.

The expression of opioid receptors on a cell indicates that it is capable of reactivity to opioid exposure. Therefore, we examined the expression of MOR in cell types that were susceptible to or infected with HIV before infection with the virus. Mu opioid receptor (MOR) expression was quantified in the positive control cell line SH-5YSY, HIV-susceptible cell line TZM-bl, as well as three HIV-infected lymphocyte cells, including J-Lat GFP, ACH-2, and H9. MOR was expressed in all cell lines, as shown in Figure 1.

### 3.2. Fentanyl Enhances HIV Replication

**Figure 2:**
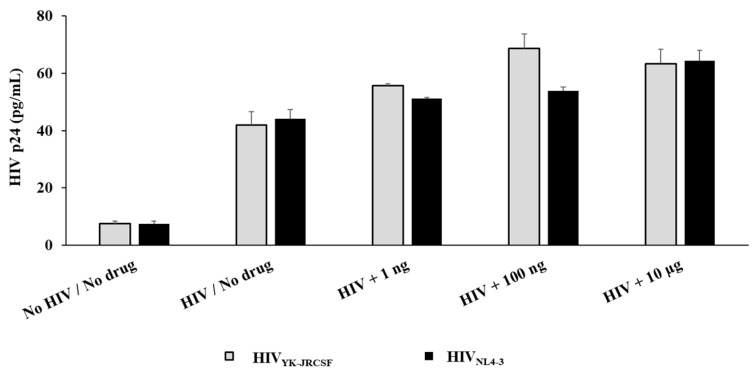
TZM-bl cells were seeded at ~2 × 10^5^ cells per well. After 24 h, cells were treated with HIV_YK-JRCSF_ and HIV_NL4-3_ at TCID_50_ of 0.5 for 1 h, rinsed with PBS three times to remove any unbound virus, and - replaced with fresh media. Fentanyl at varying concentrations was added to the respective wells and incubated. After incubation with the drug for 24 h, HIV p24 antigen (pg/mL) was quantified in culture supernatants. ANOVA for dose effect: *p* = 0.0054 for HIV_YK-JRCSF_ and *p* = 0.002 for HIV_NL4-3_.

We found expression of MOR in the HIV-susceptible and infected cells. Thus, we sought to determine whether fentanyl could modulate HIV-1 infection in vitro. The impact of fentanyl on multiple cell types was evaluated using three different concentrations: 1 ng/mL, 100 ng/mL, and 10 μg/mL. For TZM-bl cells, fentanyl exposure resulted in increased expression of the HIV p24 protein in a dose-dependent manner compared to the no-drug condition for both HIV_YK-JRCSF_ and HIV_NL4-3_ (Figure 2).

**Figure 3:**
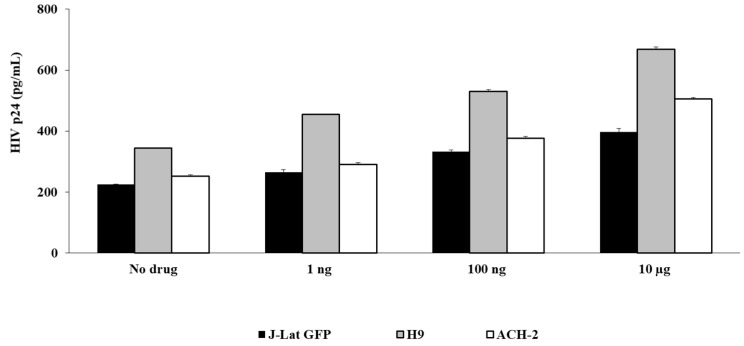
Lymphocyte cell lines ACH-2, H9, and J-Lat GFP were seeded at ~2 × 10^5^ cells per well. Fentanyl at three different concentrations was added. After incubation with the drug for 24 h, HIV p24 protein (pg/mL) was quantified in culture supernatant. ANOVA for dose effect: *p* = 0.001 for J-Lat GFP, *p* = 0.0045 for ACH-2, and *p* = 0.0062 for H9.

To evaluate the effect of fentanyl in other cell types, HIV-infected lymphocyte cell lines were also incubated with fentanyl. As shown in Figure 3, fentanyl led to a significant dose-dependent increase in expression of HIV p24 protein in the J-Lat GFP, ACH-2, and H9 cell lines as well.

**Figure 4:**
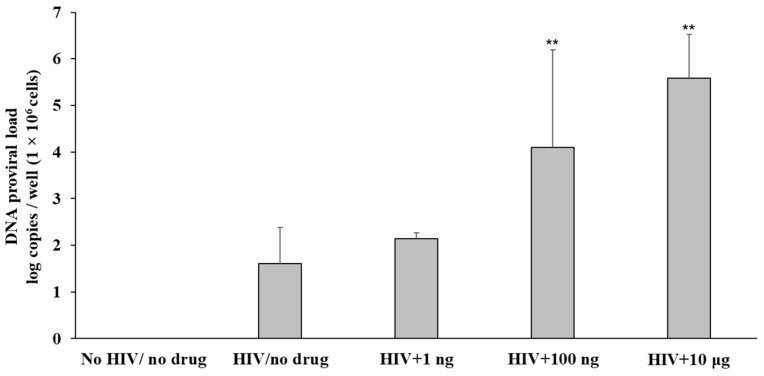
TZM-bl cells were seeded at a density of ~1 × 10^6^ cells per well. After 24 h, cells were treated with HIV_NL4-3_ for 1 h at TCID_50_ of 0.5, rinsed with PBS three times to remove any unbound virus, and replaced with fresh media. Fentanyl at varying concentrations was added to the respective wells and incubated. After incubation with the drug for 24 h, HIV proviral DNA was quantified in cells with real-time PCR based on SYBR Green detection. Error bars represent the standard deviations between the replicates. ANOVA for dose effect: *p* = 0.0006. ** *p* < 0.01.

In TZM-bl cells treated with HIV_NL4-3_ and exposed to fentanyl at varying concentrations, log copies of proviral DNA increased with dose-dependent concentration of drug compared to the virally infected but drug-naïve cells (Figure 4).

### 3.3. Fentanyl Increases HIV Co-Receptor Expression

**Figure 5:**
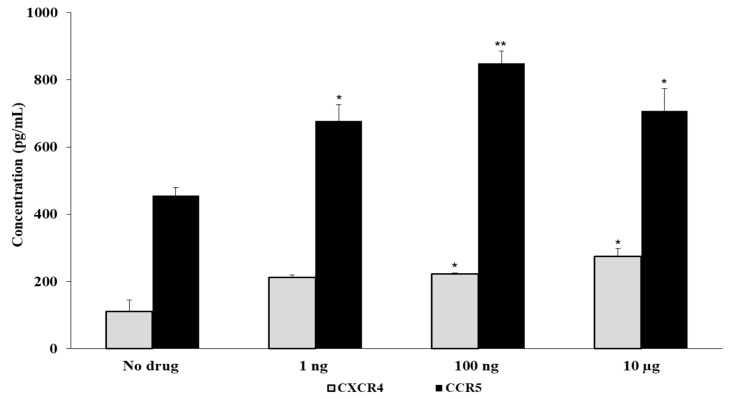
TZM-bl cells were seeded at a density of ~2 × 10^5^ cells per well. Fentanyl was added to the culture medium at three concentrations. Post incubation with the drug for 24 h, quantification of CXCR4 and CCR5 protein levels (pg/mL) was estimated with ELISA in cell culture lysates. ANOVA for dose effect: *p* = 0.0662 for CXCR4 and *p* = 0.018 for CCR5. * *p* < 0.05; ** *p* < 0.01.

Since the interaction of HIV with susceptible cells is mediated through its primary receptor (CD4) as well as chemokine co-receptors, we examined the effect of fentanyl on expression levels of CCR5 and CXCR4 proteins in uninfected cells treated with the drug. Fentanyl significantly induced the expression of CCR5 and non-significant expression of CXCR4 co-receptors in uninfected TZM-bl cells treated with fentanyl (Figure 5).

**Figure 6:**
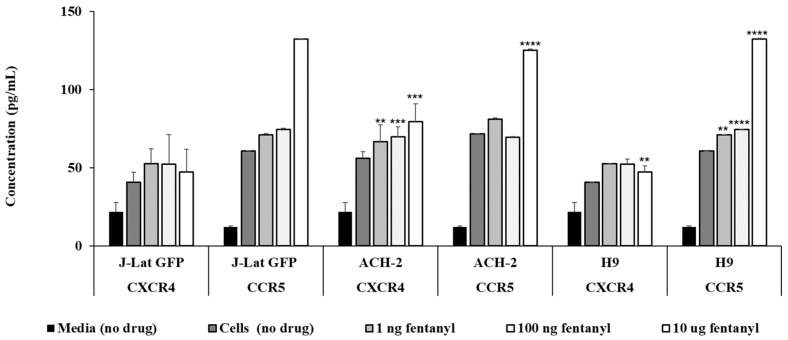
Lymphocyte cell lines were seeded at ~2 × 10^5^ cells per well. Fentanyl at three different concentrations was added. After incubation with the drug for 24 h, quantification of CXCR4 and CCR5 protein levels (pg/mL) was estimated with ELISA in cell lysates. ANOVA for dose effect on CXCR4: *p* = 0.127 for J-Lat GFP, *p* = 0.0109 for ACH-2, and *p* = 0.0026 for H9. ANOVA for dose effect on CCR5: *p* < 0.0001 for J-Lat GFP, ACH-2, and H9. ** *p* < 0.01; *** *p* < 0.001; **** *p* < 0.0001.

To further evaluate the impact of fentanyl on chemokine receptor expression, receptor levels were also quantified in HIV-infected cells exposed to fentanyl. All three HIV-infected cell lines showed significant increase in expression of CCR5. Expression of CXCR4 was significantly increased only in ACH-2 and H-9 cells. (Figure 6).

### 3.4. Fentanyl Alters Cell Viability

**Figure 7:**
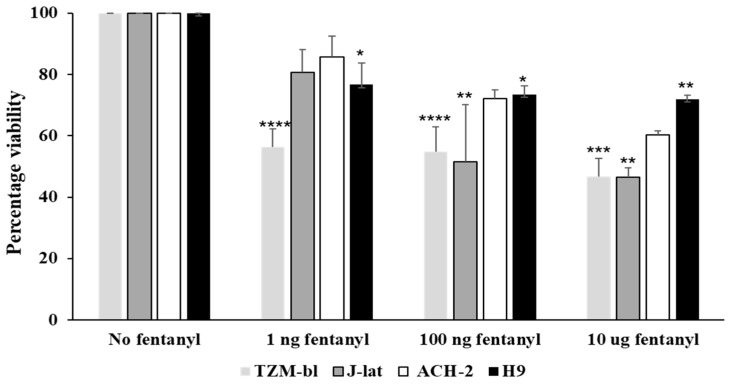
Lymphocyte cell lines were seeded at 5 × 10^4^ cells per well. Fentanyl was added to culture medium after 24 h. Post 24 h of incubation, the potential toxicity was evaluated with the MTT assay. ANOVA for dose effect: *p* = 0.018 for TZM-bl, *p* = 0.0004 for J-Lat GFP, *p* = 0.0038 for ACH-2, and *p* = 0.52 for H9. * *p* < 0.05; ** *p* < 0.01; *** *p* < 0.001; **** *p* < 0.0001.

To determine the impact of fentanyl on cell viability, HIV-susceptible and HIV-infected cell lines were treated with three different concentrations (1 ng/mL, 100 ng/mL, and 10 ug/mL) of fentanyl for 24 h. A dose-dependent decrease in cell viability with increasing concentration of fentanyl was noted in all cell types tested, but there was a minimal effect on viability for ACH-2 and H9 cell lines (Figure 7). The cells were ≥60% viable even with highest dose of fentanyl (10 ug/mL) in ACH-2 and H9 cell lines.

### 3.5. Fentanyl Enhances HIV Replication in Primary PBMC Derived T Cells

**Figure 8:**
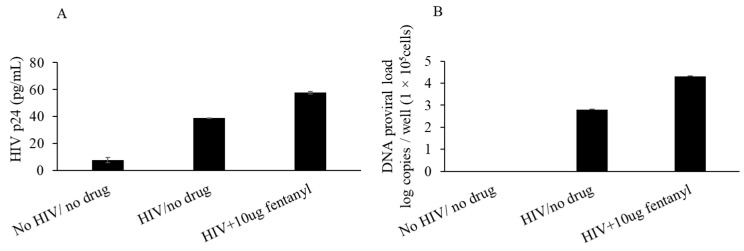
PBMC derived T cells were seeded at ~1 × 10^5^ cells per well. Cells were infected with HIV_NL4-3_ at MOI of 1 for 2 h, rinsed with RPMI + 10% FBS + 1% antibiotics (Pen/Strep) + 1% glutamine three times to remove any unbound virus and -replaced with fresh media. Fentanyl at concentration of 10 ug/mL was added to the respective wells and incubated. HIV p24 antigen expression was estimated from the cell culture supernatant (**A**), and HIV proviral DNA was quantified in cells with real-time PCR based on SYBR Green I detection on day 3 (**B**). Error bars represent the standard deviations between the replicates.

We further tested the effect of fentanyl on HIV-1 replication in CD4^+^ T cells that are the primary targets of HIV. PBMCs were isolated from peripheral blood of normal human donors, and CD4^+^ T cells were purified. Activated CD4^+^ T cells were infected with HIV_NL4-3_ and treated with fentanyl after infection. Cells treated with fentanyl showed enhanced HIVp24 expression compared to untreated ones (Figure 8A). The finding was also consistent with increase in HIV-1 replication detected by proviral DNA in the presence of fentanyl (Figure 8B).

### 3.6. Dysregulation of miRNA Profile by Fentanyl

**Figure 9:**
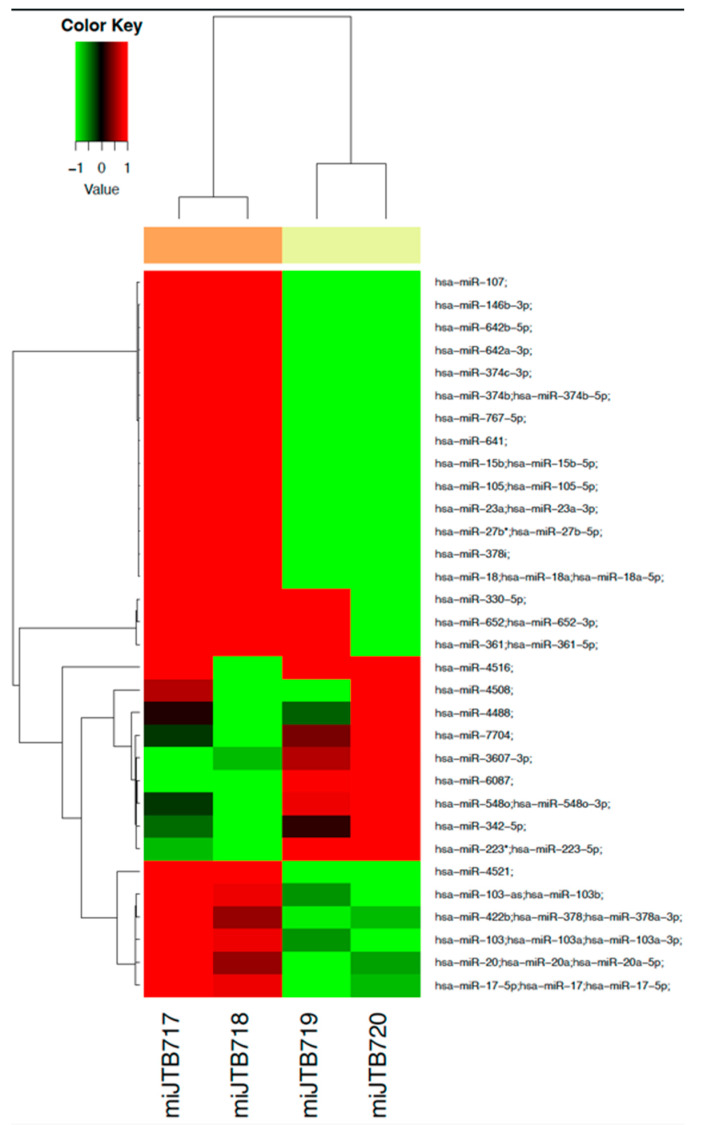
Heatmap of microRNAs that are differentially expressed in ACH-2 cell line in the presence of fentanyl versus ACH-2 without fentanyl (*p* < 0.2). The number of significant genes is 32. According to the color key at the top of the heat-map graph with dendrogram, the data are further represented as relative upregulation (red)/downregulation (green). * in hsa-miR-27b*; hsa-miR-27b-5p and hsa-miR-223*; hsa-miR-223-5p denotes the **5-prime** strand and it is less expressed than the 3-prime. (miJTB17 and miJTB18: replicates of control cells; miJTB19 and miJTB20: replicates of cells treated with 10 μg/mL of fentanyl).

Total miRNA isolated from the ACH-2 cell line treated with or without fentanyl for 24 h was used to generate a comprehensive miRNA expression profile. Comparing the fentanyl-treated versus with untreated conditions, there were 32 miRNAs differentially expressed, with *p*-value < 0.2 as shown in Figure 9.

A list of miRNAs with their *p*-values and fold changes is provided in Supplementary Table S1. Of those miRNAs previously shown to impact HIV, eight mRNAs—miR-15, miR-146b, miR-378, miR-223-3p, miR-27b, miR-17, miR-3607-3p, and miR-4516—were differentially expressed in ACH-2 cells treated with fentanyl. A list of microRNAs that may play a role in HIV pathogenesis is provided in Supplementary Table S2. MicroRNAs that are differentially expressed with *p*-value < 0.2 are denoted by ⚫️.

### 3.7. Fentanyl Alters the Cellular Transcriptome

**Figure 10:**
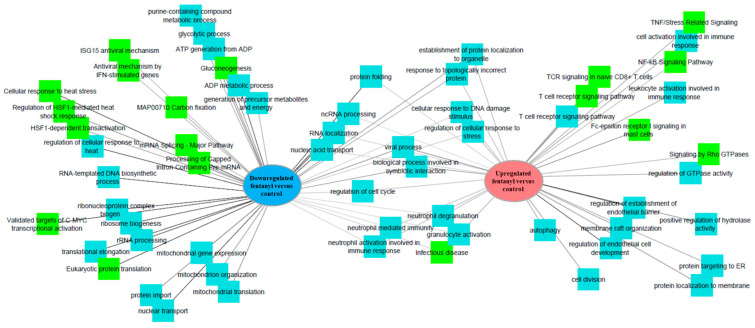
RNAseq analysis of genes that are upregulated (red) or downregulated (blue) in the ACH-2 cell line in the presence/absence of fentanyl.

To further understand the role of fentanyl in HIV pathogenesis, the ACH-2 cell line was utilized to characterize the effect of fentanyl on cellular gene expression. Differential analysis identified 1134 transcripts, with 619 up- and 515 downregulated transcripts in fentanyl-treated cells compared to untreated ones. Biological processes and pathways related to these genes are presented in Figure 10.

**Figure 11:**
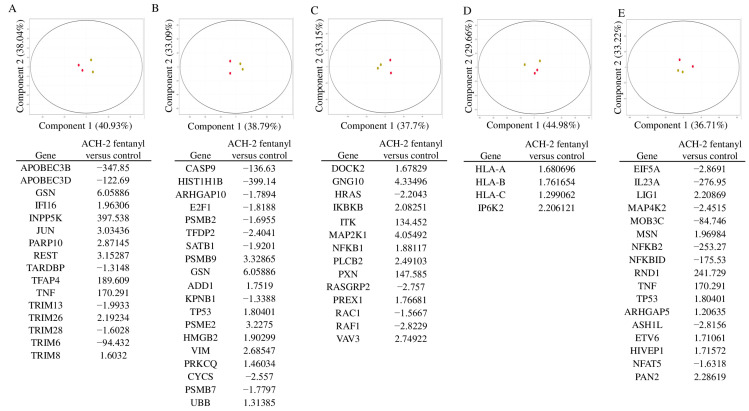
Principal component analysis of the expression of mRNA in ACH-2 cells in the presence/absence of fentanyl: (**A**) antiviral, (**B**) cell death, (**C**) chemokine, (**D**) interferon, and (**E**) NFκB signaling genes that are significantly differentially expressed (red = no fentanyl, and green = fentanyl-treated).

Candidate gene analysis identified 702 candidate genes involved in antiviral response, cell death or apoptosis, chemokine signaling, interferon response, and NFkB signaling (gene lists built and consolidated in gataca.cchmc.org). Of these 702 candidate genes, 49 were differentially regulated in treated ACH-2 cells (*p*-value < 0.05 with moderated *t*-test; Supplementary Table S3—labeled in green), with 21 more indicating a trend toward significance (*p* < 0.1 is labelled in red). As shown in Figure 11, 16 antiviral genes were significantly differently expressed in fentanyl-treated ACH-2 cells, including downregulation of *APOBEC3B, APOBEC3D, TARDBP, TRIM13, TRIM28,* and *TRIM6* and upregulation of *GSN, IFI16, INPP5K, JUN, PARP10, REST, TFAP4, TNF, TRIM26,* and *TRIM8* (Figure 11A). For cell-death pathways, ten genes were significantly downregulated (*CASP9, ARHGAP10, E2F1, HIST1H1B, PSMB2, SATB1, CYCS, PSMB7, KPNB1*, and *TFDP2*), and nine genes were upregulated (*PRKCQ, ADD1, GSN, HMGB2, PSMB9, PSME2, TP53, UBB,* and *VIM*) in fentanyl-treated ACH-2 cells (Figure 11B). Fentanyl treatment induced expression of 14 chemokine signaling genes: *HRAS, RAC1, RAF1,* and *RASGRP2* were downregulated, while *DOCK2, GNG10, IKBKB, ITK, MAP2K1, NFKB1, PLCB2, PREX1, VAV3,* and *PXN* were significantly upregulated, as shown in Figure 11C, with fold changes ranging from 1.6 to 147. For interferon signaling genes, *HLA-A, HLA-B, HLA-c*, and *IP6K2* were upregulated, with no genes significantly downregulated (Figure 11D). Finally, 17 genes involved in NFkB signaling were differentially expressed. *ASH1L, EIF5A, IL23A, MAP4K2, MOB3C, NFKB2, NFAT5*, and *NFKBID* were downregulated, while *ARHGAP5, ETV6, HIVEP1, LIG1, MSN, PAN2, RND1, TNF*, and *TP53* were upregulated (Figure 11E). As a result of fentanyl exposure, several genes show an upregulation and differential expression, suggesting a significant effect on transcriptional regulation and immune response. Thus, fentanyl augments cell death and activates antiviral, chemokine, interferon, and NFkB signaling processes leading to suppression of the immune system.

## 4. Discussion

Since 2000, the number of opioid-related overdose deaths has increased steadily. With the increased use of synthetic opioids (mainly fentanyl and fentanyl analogs), the death toll reached an all-time high in 2021 [49]. The US is facing a major public health concern due to the opioid epidemic, which can be linked to HIV infections [50,51,52,53,54]. Sharing needles, syringes, or other drug injection materials directly increases the risk of HIV infection in people with opioid use disorder (OUD) [55,56,57,58]. For instance, Degenhardt et al. reported that among people who inject the drug (PWID), 17.8% live with HIV, 52.3% are HCV-seropositive, and the HBV surface antigen is positive in 9.1% [59]. People with drug addiction are at risk of exacerbating HIV-related comorbidities [60,61]. Furthermore, compared to uninfected people, long-term opiate use increases the risk of death in HIV-positive people [62,63]. As a result, it is critical to understand the link between opioid use and increased HIV infectivity to prevent the spread of HIV and its deleterious effects.

Several drugs of abuse are known to promote viral replication by suppressing innate immune responses [8,14]. HIV expression is increased by endogenous opioid peptides. For example, in microglia, endorphin increased viral protein synthesis and LTR activation [64]. Endomorphin-1 also increased HIV expression in mixed glial/neuronal and microglial cell cultures [65], whereas dynorphin promoted HIV expression in fetal brain co-cultures [66].

Expression of HIV was increased in the presence of morphine in co-cultures of promonocytes and fetal brain cells, as well as primary Kupffer cell cultures [24,26]. Prottengeier et al. found that morphine stimulated viral reactivation in latently infected cells [67]. HIV replication increased in peripheral blood lymphocytes and T cell lines in vitro upon withdrawal of morphine [68] and antagonized viral inhibitory factors in macrophages [69]. Morphine has been shown to increase HIV expression in blood monocyte-derived macrophages and neonatal macrophages, while decreasing anti-HIV microRNA expression, upregulating chemokine receptor expression, and inhibiting interferons (IFN), as well as IFN-inducible genes and regulators of the Janus kinase signal transducer and activator of transcription (JAK–STAT)-signaling pathway [20,21,49,70].ye

The semi-synthetic opioid heroin inhibits anti-HIV microRNAs in macrophages, promoting HIV expression [71]. Prottengeier et al. reported HIV reactivation in latently infected lymphocytes by heroin in vitro [67]. MicroRNA expression was also altered in peripheral blood mononuclear cells (PBMCs) and/or macrophages isolated from heroin-dependent individuals [72]. Buprenorphine was found to increase in vitro infection of PBMC with an HIV reporter virus [73]. The synthetic opioid methadone was found to promote HIV replication in fetal microglia and blood monocyte-derived macrophages by increasing the expression of CCR5 and reducing the expression of interferon, interferon stimulated genes, and anti-HIV microRNAs [74,75].

The US Drug Enforcement Administration has found counterfeit pills ranging from 0.02 to 5.1 milligrams (more than twice the lethal dose) of fentanyl per tablet. The estimated lethal dose of fentanyl in humans is 2 mg [76]. Our unpublished data collected from persons reporting to the University of Cincinnati Medical Center with HIV and opioid overdose demonstrate that blood levels of fentanyl associated with opioid overdose ranges from 1 to 25 ng/mL. Others have reported that post-mortem blood fentanyl concentration ranges from 7 to 97 ng/mL [77]. Another study by Wu et al. reported that median concentrations of fentanyl in urine and serum or plasma samples were 34 ng/mL and 10.5 ng/mL, respectively [78].

We previously reported that fentanyl increased expression of hepatitis C virus and hepatitis B virus in hepatocyte cell lines. Fentanyl also differentially regulated genes involved in the antiviral response, chemokine signaling, NFkB signaling, apoptosis, and cell death in hepatocyte cell lines [79]. These findings are supported by other studies demonstrating that opioid receptors are expressed in the liver and are important mediators of liver disease progression [80,81]. Similarly, hepatic stellate cells express several opioid receptors and contribute significantly to hepatic fibrosis [82,83]. Other in vitro studies have shown that morphine, heroin, and methamphetamine increase HCV replication [84,85,86,87]. Several research studies have proven that drugs of abuse consisting of cocaine, methamphetamine, tetrahydrocannabinol, and alcohol elevate the expression of the CCR5 and/or CXCR4 co-receptors [88,89,90,91,92,93]. The levels of chemokine co-receptors have also been found to increase with opioids such as morphine and methadone [21,22,74,94]. Increased expression of chemokine co-receptor in the presence of fentanyl is particularly intriguing in light of data showing that MOR activation leads to the heterologous desensitization of chemokine receptors [95]. We recently also proved that fentanyl induced HIV replication and chemokine co-receptor expression in human neuroblastoma cell line SH-SY5Y, resulting in decrease in TLR9 expression [96]. Yet another study by Yan J et al. showed that HIV replication and reactivation was enhanced by fentanyl in macrophages and Jurkat C11 cells [97]. The interaction of opioid receptors with chemokine receptors may have important implications for HIV pathogenesis.

Similar to the findings reported here in lymphocyte cell lines, fentanyl induces apoptosis in peripheral blood lymphocytes and human umbilical cord mononuclear cells [98]. In addition to changing the phenotype of T lymphocytes, fentanyl reduces cell proliferation, triggers apoptosis, and decreases proinflammatory cytokines [99]. However, ours is the first study to evaluate the effect of fentanyl on HIV expression and dysregulation of host cellular pathways. In addition to the impact of fentanyl on HIV replication, our study found that fentanyl promotes chemokine receptor CCR5 expression in a dose-dependent manner in all cell types and significantly enhances the expression of CXCR4 in ACH-2 and H9 cell lines. Altered or enhanced expression of chemokine receptors by various cells by fentanyl may significantly enhance the ability of HIV pathogenesis. Thus, fentanyl may facilitate enhanced viral infection by increasing the availability of HIV-1 co-receptor. Our study included the effects of fentanyl on lymphocytes as well as TZM-bl cells, which are adherent, non-immunological cells engineered to express the HIV co-receptors CD4, CCR5, and CXCR4. This cell type is crucial for the investigation of fundamental HIV biology, drug screens, and, more recently, clinical studies of HIV persistence [34]. RNA sequencing analysis of fentanyl-treated ACH-2 cells showed multiple differentially regulated genes indicating fentanyl-driven augmentation of cell death, antiviral response, chemokine, interferon, and NFkB signaling in the HIV-infected ACH-2 cell line. Fentanyl also led to dysregulation of multiple transcriptional factors that are involved in viral replication and pathogenesis. T-cell receptor signaling was found to be upregulated in ACH-2 cells treated with fentanyl. The stimulation of TCR by fentanyl could lead to the activation of T cells and the expression of viral genes via transcription factors such as NFkB. Fentanyl also downregulated various transcriptional factors, including interferon stimulating genes, which inhibit antiviral signaling and modulate immune responses in ACH-2 cells.

Limitations of the current study design should be considered. First, the experiments describe an acute, one-time exposure to fentanyl, and the long-term impact of fentanyl on viral replication is unknown. Second, all studies were performed in vitro with lab-adapted HIV isolates; thus, complementary studies on HIV replication and cellular gene expression are warranted in individuals with HIV infection and fentanyl exposure. Third, while there were several miRNAs that were differentially expressed [100,101,102,103,104,105,106,107,108,109,110,111,112,113,114,115,116,117,118,119,120,121,122,123,124,125,126,127,128], none of these differences were statistically significant, and this analysis was limited to a single cell type. Thus, further studies are needed to explore how viral infections and synthetic opioids synergize to regulate miRNA expression levels.

## 5. Conclusions

The opioid epidemic continues to pose a significant global health problem. More studies are needed to examine how fentanyl contributes to or limits HIV pathogenesis. Our in vitro data indicates the possibility that fentanyl induces chemokine co-receptor expression and increases HIV replication. Further, precise mechanisms by which fentanyl impairs HIV infection must also be determined through in vitro and ex vivo studies. Increased knowledge on virus-drug interactions may ultimately lead to the improvement of existing therapeutic interventions or the development of novel therapeutic approaches for this difficult-to-treat population.

## Data Availability

All data generated during and/or analyzed during the current study are included in this article. The RNAseq and miRNAseq data are available within GEO and have been assigned the numbers GSE216124 and GSE216044, respectively. All other relevant data are within the paper and its supporting information files.

## Supplementary Materials

The following supporting information can be downloaded at: https://www.mdpi.com/article/10.3390/v15041027/s1, Table S1: List of miRNAs with their p-value and fold change, Table S2: List of microRNAs that may play a role in HIV pathogenesis, Table S3: List of differentially regulated genes in fentanyl treated ACH-2 cells.
