# Supplementary material for: The Synthetic Opioid Fentanyl Increases HIV Replication and Chemokine Co-Receptor Expression in Lymphocyte Cell Lines
Source: Viruses. 2023 Apr 21;15(4):1027. doi: 10.3390/v15041027 (PMC10145664; doi:10.3390/v15041027)
Supplement: Supplementary file 1 [file viruses-15-01027-s001.zip › viruses-2341782-supplementary.pdf]

Table S1. List of miRNAs with their *p*-value and fold change.

Gene ID	Symbol	Name	Base Mean	Base Mean Con1	Base Mean Fent1	Fold Change	log2Fold Change	pval	padj	ACH-2 Control Replicate-1	ACH-2 Control Replicate-2	ACH-2 with Fentanyl Replicate-1	ACH-2 with Fentanyl Replicate-2
MIMAT0023712	hsa-miR-6087;	hsa-miR-6087;	36	7.7	64.6	7.3	2.9	0.0	1.0	5193.9	6822.4	28043.5	58470.4
MIMAT0030019	hsa-miR-7704;	hsa-miR-7704;	82	20.7	142.9	5.8	2.5	0.0	1.0	25969.4	6001.7	41948.7	137893.2
MIMAT0019058	hsa-miR-4521;	hsa-miR-4521;	14	25.3	2.9	0.1	-3.2	0.0	1.0	18178.6	14752.4	1207.6	2035.7
MIMAT0000102	hsa-miR-105;hsa-miR-105-5p;	hsa-miR-105;hsa-miR-105-5p;	3	5.6	0.0	0.0	-6.0	0.0	1.0	2709.9	4195.2	0.0	0.0
MIMAT0000104	hsa-miR-107;	hsa-miR-107;	3	6.6	0.0	0.0	-6.3	0.0	1.0	6548.8	1906.9	0.0	0.0
MIMAT0004570	hsa-miR-223*;hsa-miR-223-5p;	hsa-miR-223*;hsa-miR-223-5p;	21	5.8	37.1	5.6	2.5	0.0	1.0	5193.9	2392.3	24152.3	17303.6
MIMAT0000078	hsa-miR-23a;hsa-miR-23a-3p;	hsa-miR-23a;hsa-miR-23a-3p;	2	4.7	0.0	0.0	-5.7	0.0	1.0	2967.9	3341.6	0.0	0.0
MIMAT0007402	hsa-miR-103-as;hsa-miR-103b;	hsa-miR-103-as;hsa-miR-103b;	129	194.8	63.4	0.3	-1.8	0.1	1.0	130976.3	110981.1	42739.0	25313.7
MIMAT0000101	hsa-miR-103;hsa-miR-103a;hsa-miR-103a-3p;	hsa-miR-103;hsa-miR-103a;hsa-miR-103a-3p;	129	194.8	63.4	0.3	-1.8	0.1	1.0	130976.3	110981.1	42739.0	25313.7
MIMAT0019045	hsa-miR-4508;	hsa-miR-4508;	41	8.2	74.5	7.4	2.9	0.1	1.0	12220.9	2063.9	1562.8	101426.7
MIMAT0004766	hsa-miR-146b-3p;	hsa-miR-146b-3p;	1	2.8	0.0	0.0	-5.0	0.1	1.0	1652.6	1993.6	0.0	0.0
MIMAT0003882	hsa-miR-767-5p;	hsa-miR-767-5p;	1	2.5	0.0	0.0	-4.8	0.1	1.0	1129.1	1906.9	0.0	0.0
MIMAT0000075	hsa-miR-20;hsa-miR-20a;hsa-miR-20a-5p;	hsa-miR-20;hsa-miR-20a;hsa-miR-20a-5p;	68	101.5	34.5	0.3	-1.7	0.1	1.0	79940.7	47290.9	15016.4	21419.3
MIMAT0019022	hsa-miR-4488;	hsa-miR-4488;	24	6.0	41.4	5.6	2.5	0.1	1.0	8367.9	1461.9	5903.9	48518.0
MIMAT0000732	hsa-miR-422b;hsa-miR-378;hsa-miR-378a-3p;	hsa-miR-422b;hsa-miR-378;hsa-miR-378a-3p;	191	279.8	102.1	0.3	-1.6	0.1	1.0	220504.1	145131.7	53135.0	60053.7
MIMAT0022736	hsa-miR-642b-5p;	hsa-miR-642b-5p;	1	2.2	0.0	0.0	-4.6	0.1	1.0	1180.4	1594.9	0.0	0.0
MIMAT0020924	hsa-miR-642a-3p;	hsa-miR-642a-3p;	1	2.2	0.0	0.0	-4.6	0.1	1.0	1180.4	1594.9	0.0	0.0
MIMAT0000070	hsa-miR-17-5p;hsa-miR-17;hsa-miR-17-5p;	hsa-miR-17-5p;hsa-miR-17;hsa-miR-17-5p;	45	67.5	22.7	0.3	-1.7	0.1	1.0	43583.5	40044.7	10396.0	13630.5

MIMAT0000417	hsa-miR-15b;hsa-miR-15b-5p;	hsa-miR-15b;hsa-miR-15b-5p;	2	3.5	0.0	0.0	-5.3	0.1	1.0	2596.9	1993.6	0.0	0.0
MIMAT0004694	hsa-miR-342-5p;	hsa-miR-342-5p;	150	70.7	230.3	2.8	1.5	0.1	1.0	57380.1	39263.8	87293.2	177010.6
MIMAT0003311	hsa-miR-641;	hsa-miR-641;	1	2.3	0.0	0.0	-4.7	0.1	1.0	865.6	1827.4	0.0	0.0
MIMAT0003322	hsa-miR-652;hsa-miR-652-3p;	hsa-miR-652;hsa-miR-652-3p;	4	7.7	1.0	0.1	-3.0	0.1	1.0	5935.9	4594.7	1265.1	0.0
MIMAT0022735	hsa-miR-374c-3p;	hsa-miR-374c-3p;	1	2.1	0.0	0.0	-4.5	0.1	1.0	708.3	1993.6	0.0	0.0
MIMAT0000703	hsa-miR-361;hsa-miR-361-5p;	hsa-miR-361;hsa-miR-361-5p;	4	6.6	1.0	0.1	-2.8	0.1	1.0	5430.0	3189.7	1207.6	0.0
MIMAT0004955	hsa-miR-374b;hsa-miR-374b-5p;	hsa-miR-374b;hsa-miR-374b-5p;	1	2.1	0.0	0.0	-4.5	0.1	1.0	708.3	1993.6	0.0	0.0
MIMAT0019053	hsa-miR-4516;	hsa-miR-4516;	5	1.6	8.8	4.2	2.1	0.1	1.0	2749.7	0.0	3125.6	9220.6
MIMAT0005919	hsa-miR-548o;hsa-miR-548o-3p;	hsa-miR-548o;hsa-miR-548o-3p;	3	1.2	4.9	3.2	1.7	0.2	1.0	1180.4	398.7	2415.2	3053.6
MIMAT0017985	hsa-miR-3607-3p;	hsa-miR-3607-3p;	9	3.3	13.8	3.6	1.9	0.2	1.0	2077.6	2631.5	6641.9	10076.8
MIMAT0004588	hsa-miR-27b*;hsa-miR-27b-5p;	hsa-miR-27b*;hsa-miR-27b-5p;	1	1.5	0.0	0.1	-4.2	0.2	1.0	1180.4	797.4	0.0	0.0
MIMAT0000072	hsa-miR-18;hsa-miR-18a;hsa-miR-18a-5p;	hsa-miR-18;hsa-miR-18a;hsa-miR-18a-5p;	1	1.7	0.0	0.0	-4.4	0.2	1.0	1354.9	762.8	0.0	0.0
MIMAT0019074	hsa-miR-378i;	hsa-miR-378i;	1	1.7	0.0	0.0	-4.4	0.2	1.0	1484.0	835.4	0.0	0.0
MIMAT0004693	hsa-miR-330-5p;	hsa-miR-330-5p;	3	5.4	1.0	0.2	-2.5	0.2	1.0	4249.5	2791.0	1207.6	0.0

Table S2. List of microRNAs that may play a role in HIV pathogenesis. MicroRNAs that are differentially expressed with p-value < 0.2 are denoted by ●.

microRNA	Effect	References	ACH-2 in the prescence of Fentanyl
15		Su 2018 [100]	●
16		Su 2018 [100]	
17	Negative	Triboulet R 2007 [101]	●
20		Su 2018 [100]	
28		Su 2018 [100]	
31		Sung and Rice 2009 [102]	
101		Su 2018 [100]	
132	Positive	Chiang 2013 [103]	
144		Su 2018 [100]	
149	Negative	Hariharan 2005 [104]	
150		Su 2018 [100]	
155		Su 2018; Pilakka-Kanthikeel 2015 [100,105]	
155	Positive	Jin C 2017 [106]	
193		Su 2018 [100]	
198		Su 2018 [100]	
198	Negative	Sung and Rice 2009 [102]	
200		Sung and Rice 2009 [102]	
217		Kwon and Ott 2008 [107]	
221		Egana-Gorrono 2014 [108]	
326	Negative	Houzet 2012 [109]	
349		Duskova 2013[128]	
378		Su 2018 [100]	●
378	Negative	Hariharan 2005 [104]	
382		Su 2018 [100]	
503		Sung and Rice 2009 [102]	
526		Sung and Rice 2009 [102]	
888	Negative	Chen 2014 [110]	
1290	Negative	Wang 2015 [111]	
4508		Egaña-Gorroño L 2016 [113]	
4516		Asahchop 2016 [114]	●
106b		Su 2018 [100]	
125b		Su 2018; Mantri 2012[100]	
125b-5p	Negative	Huang 2007 [112]	
133b	Negative	Houzet 2012 [109]	
138-5p	Negative	Houzet 2012 [109]	
146a	Negative	Chen 2014 [110]	
146b		Su 2018 [100]	●
149-5p	Negative	Houzet 2012 [109]	
150-5p	Negative	Huang 2007; Wang 2015 [112, 111]	
155-5p	Negative	Dey 2016 [115]	
181a	Positive	Pilakka-Kanthikeel 2015 [116]	
191-5p	Negative	Zheng Y 2021 [117]	
196b	Negative	Wang 2015 [111]	
19b		Su 2018 [100]	
223-3p	Negative	Huang 2007; Wang 2015 [112, 111]	●
27a		Egana-Gorrono 2014 [108]	
27b		Egana-Gorrono 2014 [108]	●
28-5p	Negative	Huang 2007; Wang 2015 [112, 111]	
29a	Negative	Su 2018 [100]	
29a-3p	Negative	Nathans 2009 [118]	
29b		Su 2018 [100]	
29b-3p	Negative	Sun 2012 [119]	
3146a-5p		Reynoso 2014 [120]	
3162_3p		Huang 2018 [121]	
324-5p		Su 2018 [100]	
33a-5p		Su 2018 [100]	
341-3p		Su 2018 [100]	
342-3p		Su 2018 [100]	
34a		Kwon and Ott 2008 [107]	
34c-5p	Positive	Su 2018 [100]	
3607-3p		Chang ST 2013 [122]	●
382-5p	Negative	Huang 2007; Wang 2015 [112, 111]	
423-3p	Negative	Whisnant 2013 [123]	
615-3p		Su 2018 [100]	
92a-3p	Negative	Houzet 2012 [109]	
99a		Sung and Rice 2009 [102]	
H1-HIV-encoded		Kaul 2009 [124]	
H3-HIV-encoded		Zhang 2014 [125]	

let-7e	Su 2018 [100]	
N367-HIV-encoded	Omoto 2004 [126]	
TAR-3p-HIV-encoded	Harwig A [127]	
**Total number correlated**		**8**
**Total number of significant genes detected**		**32**

Table S3. List of differentially regulated genes in fentanyl treated ACH-2 cells. The list of genes that are significantly regulated are labeled in green (p-value < 0.05 by moderated t-test; –), with 21 more indicating a trend toward significance are labelled in red (p-value < 0.1).

S.No	Gene	p ([Ctrl] vs [Fent])	Regulation	Fold change
		**Antiviral**		
1	ADAR	0.88652	down	−1.0406
2	APCS	1	down	−1
3	APOBEC3A	0.37358	up	14.4346
4	APOBEC3B	1.08E-05	down	−347.85
5	APOBEC3C	0.18229	up	2.21937
6	APOBEC3D	9.60E-04	down	−122.69
7	APOBEC3F	0.37358	up	17.6086
8	APOBEC3G	0.63923	up	1.22118
9	APOBEC3H	1	down	−1
10	BST2	0.63858	down	−1.5433
11	BTBD17	1	down	−1
12	C19orf66	0.79871	down	−1.0916
13	CCNK	0.988	down	−1.0087
14	CEACAM1	1	down	−1
15	CHMP3	0.79257	up	1.14245
16	EIF2AK2	0.21957	up	2.11167
17	EIF2AK4	0.82777	up	1.09961
18	FAM111A	0.25928	up	1.23637
19	FBLN1	1	down	−1
20	FCN1	1	down	−1
21	FCN3	1	down	−1
22	GSN	0.01065	up	6.05886
23	HDAC1	0.44975	up	1.20977
24	HMGA2	1	down	−1
25	IFI16	0.09172	up	1.96306
26	IFIT1	0.93228	down	−1.3167
27	IFITM1	0.52872	up	1.25463
28	IFITM2	0.68051	down	−1.3616
29	IFITM3	1	down	−1
30	IFNA2	1	down	−1
31	IFNB1	1	down	−1
32	IFNL3	1	down	−1
33	ILF3	0.43483	down	−1.3283
34	INPP5K	3.43E-05	up	397.538
35	ISG15	0.73606	up	1.15973
36	ISG20	1	down	−1
37	JUN	0.08212	up	3.03436
38	LTF	1	down	−1
39	MAVS	0.142	up	1.35803
40	MBL2	1	down	−1
41	MID2	0.37358	up	6.34281
42	MX1	0.37358	down	−11.278
43	NBN	0.505	up	1.2004
44	OAS1	0.37358	up	26.9376
45	OAS3	0.92876	up	1.04212
46	OASL	1	down	−1
47	PARP10	0.06804	up	2.87145
48	PLSCR1	0.27056	up	34.6392
49	PML	0.5475	down	−1.0418
50	POU2F3	1	down	−1
51	PROX1	1	down	−1
52	PTX3	1	down	−1
53	RAD50	0.91593	up	1.04654
54	REST	0.05405	up	3.15287
55	RNASEL	0.48237	down	−1.2191
56	RSAD2	0.37358	down	−8.8418

57	SLPI	1	down	−1
58	SNX3	0.33192	up	1.33217
59	SP100	0.44692	up	1.55679
60	SRPK1	0.25565	down	−1.3319
61	SRPK2	0.62493	up	1.19935
62	STAT1	0.38021	up	1.44865
63	TARDBP	0.00111	down	−1.3148
64	TFAP4	7.93E-04	up	189.609
65	TNF	1.10E-05	up	170.291
66	TNIP1	0.27302	down	−1.4924
67	TRIM10	1	down	−1
68	TRIM11	0.89114	up	1.10269
69	TRIM13	0.04278	down	−1.9933
70	TRIM14	0.77569	down	−1.1578
71	TRIM15	1	down	−1
72	TRIM21	0.47329	up	10.3722
73	TRIM25	0.41929	down	−1.3766
74	TRIM26	0.03591	up	2.19234
75	TRIM27	0.32817	down	−1.9548
76	TRIM28	0.0063	down	−1.6028
77	TRIM31	1	down	−1
78	TRIM32	0.83858	up	2.07532
79	TRIM35	0.12271	up	1.58826
80	TRIM5	0.31635	up	24.9882
81	TRIM56	0.53266	up	1.89347
82	TRIM59	0.2733	up	1.61728
83	TRIM6	4.50E-08	down	−94.432
84	TRIM62	0.36432	up	8.59365
85	TRIM8	0.02798	up	1.6032
86	VAPA	0.47273	down	−1.2236
87	VAPB	0.4507	down	−1.2851
88	ZC3H12A	0.98093	up	1.08089
89	ZC3HAV1	0.70343	up	1.12694
90	ZNF639	0.73133	up	1.15464
		**Cell death**		
91	BMX	1	down	−1
92	BBC3	1	down	−1
93	DAPK3	0.96369	down	−1.2019
94	DSG3	1	down	−1
95	DSG1	1	down	−1
96	MAPT	1	down	−1
97	BAX	0.94988	down	−1.3581
98	DSP	1	down	−1
99	HIST1H1A	1	down	−1
100	PSMB10	0.38151	down	−22.99
101	DAPK2	1	down	−1
102	CD14	1	down	−1
103	TICAM2	1	down	−1
104	UNC5A	1	down	−1
105	DCC	1	down	−1
106	BMF	1	down	−1
107	SFN	1	down	−1
108	PKP1	0.37358	down	−6.5257
109	BAD	0.90015	up	1.76439
110	TP73	1	down	−1
111	CASP9	1.93E-07	down	−136.63
112	HIST1H1E	0.37358	down	−20.122
113	DFFB	0.37358	down	−9.3878
114	PRKCD	0.37358	down	−10.09
115	HIST1H1D	1	down	−1
116	CDH1	1	down	−1
117	TNFRSF10A	0.37358	down	−12.091
118	UNC5B	1	down	−1
119	LY96	0.37358	down	−30.155
120	PTK2	1	down	−1
121	FASLG	1	down	−1
122	TLR4	1	down	−1
123	GAS2	1	down	−1
124	TP63	1	down	−1
125	GZMB	1	down	−1
126	PSMD9	0.4026	down	−16.658

127	HIST1H1B	4.71E-07	down	−399.14
128	PSMB11	1	down	−1
129	TJP1	1	down	−1
130	TRADD	0.99207	up	1.03748
131	PPP1R13B	0.47696	down	−1.3227
132	ARHGAP10	0.00198	down	−1.7894
133	RIPK1	0.40118	down	−2.4836
134	TICAM1	0.30464	up	14.5132
135	TLR3	0.37358	up	10.1676
136	BCL2L11	0.4009	up	14.5325
137	TJP2	0.37499	down	−11.571
138	FAS	0.89056	down	−1.0923
139	RIPK3	0.3078	up	18.1291
140	FADD	0.62923	up	1.35857
141	LMNA	0.32631	up	14.6425
142	TNFSF10	0.56597	down	−1.2228
143	APAF1	0.48626	up	1.25049
144	PSMD5	0.19088	down	−2.2053
145	BIRC3	0.9337	down	−1.0856
146	PSMF1	0.32187	up	1.31185
147	APC	0.62561	down	−1.1818
148	DNM1L	0.29106	down	−1.7016
149	AKT3	0.48109	down	−1.3519
150	BCL2L1	0.43407	down	−10.823
151	PPP3CC	0.44018	down	−10.288
152	BAK1	0.11794	down	−1.7279
153	DAPK1	0.10725	up	2.41407
154	DFFA	0.68044	down	−1.1944
155	TRAF2	0.35633	down	−1.3088
156	KPNA1	0.86286	up	1.07722
157	OCLN	0.9638	up	1.04321
158	PLEC	0.12833	up	4.4146
159	E2F1	0.01591	down	−1.8188
160	AKT2	0.63871	down	−1.2328
161	MAPK8	0.79763	down	−1.1004
162	TNFRSF10B	0.56555	up	1.81491
163	BCL2	0.70118	up	1.4075
164	PRKCQ	0.07653	up	1.46034
165	PSMD12	0.76631	up	1.29394
166	BID	0.1994	up	1.12442
167	CFLAR	0.23041	up	26.4385
168	PSME4	0.38025	down	−1.1343
169	PSMB2	0.01436	down	−1.6955
170	BIRC2	0.41707	up	1.32096
171	MLKL	0.38089	down	−20.461
172	DIABLO	0.44222	up	16.2425
173	PSMA8	0.31197	up	22.5945
174	XIAP	0.10283	up	1.61093
175	TFDP2	0.04368	down	−2.4041
176	CASP7	0.33028	up	26.6412
177	DSG2	0.66176	down	−1.1508
178	HIST1H1C	0.37358	up	23.0742
179	APPL1	0.84681	down	−1.0112
180	PSMA5	0.29868	down	−1.2138
181	PAK2	0.30117	down	−1.2952
182	OPA1	0.23671	up	1.65632
183	TP53BP2	0.99204	up	1.01223
184	STK26	0.84973	up	1.13228
185	CLSPN	0.61654	down	−1.1331
186	STK24	0.35418	down	−1.2178
187	PSMD1	0.23009	down	−1.4371
188	CYCS	0.08556	down	−2.557
189	PPP3R1	0.74455	down	−1.177
190	TFDP1	0.12466	down	−2.1248
191	SATB1	0.04668	down	−1.9201
192	YWHAG	0.28338	down	−1.6428
193	PMAIP1	0.13778	up	1.31125
194	PSMC2	0.21965	up	1.2805
195	CASP6	0.68342	down	−1.0687
196	PSMA3	0.34038	down	−1.8195
197	OMA1	0.71921	up	1.45093
198	PSMD11	0.81248	down	−1.0722
199	MAGED1	0.23664	down	−1.3282

200	PSMB9	3.42E-04	up	3.32865
201	PSMB5	0.33331	down	−1.8761
202	GSN	0.01065	up	6.05886
203	PSME3	0.55071	down	−1.3487
204	PSME1	0.83179	up	1.1909
205	NMT1	0.27353	up	1.73024
206	YWHAH	0.13498	down	−1.8072
207	DBNL	0.57685	up	1.09583
208	PSMC4	0.71156	down	−1.1718
209	ADD1	0.00204	up	1.7519
210	PSMC6	0.54975	up	1.20034
211	AKT1	0.13049	down	−1.3801
212	PSMD7	0.72164	up	1.03231
213	ROCK1	0.14633	up	1.11912
214	CASP3	0.32313	down	−1.2895
215	PSMA2	0.71828	down	−1.1299
216	FNTA	0.54916	down	−10.037
217	PSMD2	0.16635	down	−1.5651
218	PSMD3	0.39265	down	−2.3255
219	ACIN1	0.24077	down	−2.072
220	PSMD13	0.42861	down	−1.7164
221	PSMB1	0.24434	down	−1.5155
222	PSMC1	0.36562	down	−1.3956
223	PSMA1	0.8486	down	−1.0649
224	PSMB7	0.0752	down	−1.7797
225	PSMD14	0.87231	up	1.05948
226	CTNNB1	0.16543	up	1.81613
227	CASP8	0.55977	up	1.72412
228	SPTAN1	0.30384	up	1.71536
229	PSMC3	0.12212	down	−2.1907
230	PSMB8	0.9535	down	−1.0191
231	H1F0	0.19967	up	2.73541
232	PSMB6	0.49524	down	−1.248
233	PSMD10	0.17213	up	2.132
234	PSMD8	0.38639	up	1.52298
235	BCAP31	0.75058	up	1.26663
236	UBC	0.31332	down	−1.3998
237	PSMD4	0.24035	up	1.435
238	PSMA4	0.5224	down	−1.1716
239	KPNB1	2.58E-04	down	−1.3388
240	YWHAB	0.88491	down	−1.0361
241	PSMD6	0.13297	up	1.38161
242	TP53	0.04771	up	1.80401
243	PSMC5	0.72451	down	−1.1241
244	PSMB4	0.28064	down	−1.5367
245	LMNB1	0.37105	up	1.18916
246	PSMA7	0.2934	down	−1.3646
247	YWHAQ	0.15238	down	−1.2362
248	PSMA6	0.21145	down	−1.4608
249	YWHAE	0.16999	down	−1.4784
250	PSME2	0.04133	up	3.2275
251	DYNLL1	0.80404	down	−1.0631
252	HMGB1	0.97706	up	1.00216
253	YWHAZ	0.23433	up	1.31521
254	UBA52	0.44288	up	1.06547
255	HMGB2	0.00499	up	1.90299
256	RPS27A	0.77323	down	−1.0158
257	VIM	0.00222	up	2.68547
258	UBB	0.0568	up	1.31385
		**Chemokine**		
259	ADCY1	1	down	−1
260	ADCY2	1	down	−1
261	ADCY3	0.92067	up	1.0868
262	ADCY4	0.37358	up	8.49834
263	ADCY5	1	down	−1
264	ADCY6	0.37358	down	−6.2535
265	ADCY7	0.30675	up	14.649
266	ADCY8	1	down	−1
267	ADCY9	0.37358	down	−8.2008
268	AKT1	0.13049	down	−1.3801
269	AKT2	0.63871	down	−1.2328
270	AKT3	0.48109	down	−1.3519
271	ARRB1	0.88238	down	−1.0982

272	ARRB2	0.1414	down	−1.9308
273	BCAR1	1	down	−1
274	BRAF	0.27861	up	2.3275
275	CCL1	1	down	−1
276	CCL11	1	down	−1
277	CCL13	1	down	−1
278	CCL17	1	down	−1
279	CCL19	1	down	−1
280	CCL2	1	down	−1
281	CCL20	1	down	−1
282	CCL21	1	down	−1
283	CCL22	1	down	−1
284	CCL24	1	down	−1
285	CCL25	1	down	−1
286	CCL26	1	down	−1
287	CCL27	1	down	−1
288	CCL28	1	down	−1
289	CCL7	1	down	−1
290	CCL8	1	down	−1
291	CCR1	0.37358	down	−9.7138
292	CCR10	1	down	−1
293	CCR2	1	down	−1
294	CCR3	1	down	−1
295	CCR4	0.93721	up	1.44873
296	CCR5	1	down	−1
297	CCR6	1	down	−1
298	CCR7	0.98299	up	1.07677
299	CCR8	1	down	−1
300	CCR9	1	down	−1
301	CDC42	0.75086	up	1.1594
302	CHUK	0.50412	down	−1.2536
303	CRK	0.88691	down	−1.0691
304	CRKL	0.8074	down	−1.0713
305	CX3CL1	1	down	−1
306	CX3CR1	0.37358	up	9.5964
307	CXCL1	1	down	−1
308	CXCL10	1	down	−1
309	CXCL11	1	down	−1
310	CXCL12	1	down	−1
311	CXCL13	1	down	−1
312	CXCL16	0.93352	up	1.34627
313	CXCL2	1	down	−1
314	CXCL3	0.37358	up	16.984
315	CXCL5	1	down	−1
316	CXCL6	1	down	−1
317	CXCL8	1	down	−1
318	CXCL9	1	down	−1
319	CXCR1	1	down	−1
320	CXCR2	1	down	−1
321	CXCR3	1	down	−1
322	CXCR4	0.52572	down	−1.1104
323	CXCR5	1	down	−1
324	CXCR6	1	down	−1
325	DOCK2	0.03907	up	1.67829
326	ELMO1	0.29805	up	1.33253
327	FGR	1	down	−1
328	FOXO3	0.16927	up	2.23194
329	GNAI1	1	down	−1
330	GNAI2	0.55328	up	1.06171
331	GNAI3	0.36895	up	1.65423
332	GNB1	0.20052	up	1.18781
333	GNB2	0.61343	down	−1.1052
334	GNB3	0.37358	down	−11.986
335	GNB4	0.53829	down	−1.2765
336	GNB5	0.75745	down	−1.08
337	GNG10	0.01155	up	4.33496
338	GNG11	1	down	−1
339	GNG12	1	down	−1
340	GNG13	1	down	−1
341	GNG2	0.11172	up	2.44759
342	GNG3	1	down	−1
343	GNG4	1	down	−1
344	GNG5	0.31003	down	−1.2821

345	GNG7	1	down	−1
346	GNG8	1	down	−1
347	GNGT1	1	down	−1
348	GNGT2	0.37358	down	−25.649
349	GRB2	0.32918	down	−1.2258
350	GRK1	1	down	−1
351	GRK2	0.83571	up	1.04019
352	GRK3	0.39898	up	1.85103
353	GRK4	1	down	−1
354	GRK5	0.47382	up	7.85783
355	GRK6	0.98655	down	−1.0135
356	GRK7	0.37358	down	−12.04
357	GSK3A	0.70985	up	1.17434
358	GSK3B	0.3232	down	−1.3657
359	HCK	1	down	−1
360	HRAS	0.03552	down	−2.2043
361	IKBKB	0.03252	up	2.08251
362	ITK	1.15E-05	up	134.452
363	JAK2	0.81695	down	−1.1811
364	JAK3	0.16216	up	2.53706
365	KRAS	0.55227	up	1.37549
366	LYN	1	down	−1
367	MAP2K1	0.01787	up	4.05492
368	MAPK1	0.29871	up	1.36281
369	MAPK3	0.27823	up	1.5037
370	NCF1	1	down	−1
371	NFKB1	0.03529	up	1.88117
372	NFKBIA	0.41133	up	14.5421
373	NFKBIB	0.60382	down	−1.2591
374	NRAS	0.35136	up	1.25179
375	PAK1	0.10659	down	−1.7687
376	PARD3	0.37993	up	14.4392
377	PF4	1	down	−1
378	PF4V1	1	down	−1
379	PIK3CA	0.87587	down	−1.0686
380	PIK3CB	0.65523	up	1.37366
381	PIK3CD	0.40677	up	1.9174
382	PIK3R1	0.16861	up	1.72703
383	PIK3R2	0.12718	up	2.14107
384	PIK3R3	0.38517	up	1.37425
385	PLCB1	0.58731	down	−4.5302
386	PLCB2	0.01148	up	2.49103
387	PLCB3	0.97927	down	−1.0951
388	PLCB4	1	down	−1
389	PPBP	1	down	−1
390	PREX1	0.07017	up	1.76681
391	PRKACA	0.13591	down	−1.6367
392	PRKACB	0.28286	up	1.34527
393	PRKACG	1	down	−1
394	PRKCB	0.35731	up	1.48241
395	PRKCD	0.37358	down	−10.09
396	PRKCZ	0.37358	down	−11.989
397	PTK2	1	down	−1
398	PTK2B	0.29674	up	3.11915
399	PXN	2.18E-04	up	147.585
400	RAC1	0.09708	down	−1.5667
401	RAC2	0.45206	up	1.41186
402	RAF1	0.06508	down	−2.8229
403	RAP1A	0.19801	up	1.21835
404	RAP1B	0.15289	down	−1.1922
405	RASGRP2	0.01276	down	−2.757
406	RELA	0.49335	down	−1.4668
407	RHOA	0.62937	down	−1.0811
408	ROCK1	0.14633	up	1.11912
409	ROCK2	0.70567	up	1.0678
410	SHC1	0.71552	up	1.35322
411	SHC2	1	down	−1
412	SHC3	1	down	−1
413	SHC4	1	down	−1
414	SOS1	0.74186	down	−1.2853
415	SOS2	0.27102	up	2.40797
416	SRC	0.53361	down	−1.2698
417	STAT1	0.38021	up	1.44865

418	STAT2	0.42189	up	1.46378
419	STAT3	0.12597	up	2.33681
420	STAT5B	0.96855	up	1.00879
421	TIAM1	0.52048	up	1.39615
422	VAV1	0.73544	down	−1.2173
423	VAV2	0.37358	up	10.6416
424	VAV3	0.0813	up	2.74922
425	WAS	0.75225	up	1.11257
426	WASL	0.35306	up	8.84513
427	XCR1	1	down	−1
		**Interferon**		
428	ADAR	0.886521	down	−1.04059
429	BST2	0.638579	down	−1.54325
430	EGR1	0.373579	up	9.043283
431	GBP2	0.373579	up	8.473002
432	HLA-A	0.014832	up	1.680696
433	HLA-B	0.096691	up	1.761654
434	HLA-C	0.097173	up	1.299062
435	HLA-E	0.103501	up	6.642726
436	HLA-F	1	down	−1
437	HLA-G	0.373579	up	19.87244
438	HLA-H	1	down	−1
439	IFI27	1	down	−1
440	IFI35	1	down	−1
441	IFI6	0.373579	up	19.75292
442	IFIT1	0.932282	down	−1.31671
443	IFIT2	1	down	−1
444	IFIT3	1	down	−1
445	IFITM1	0.528722	up	1.254626
446	IFITM2	0.680513	down	−1.36165
447	IFITM3	1	down	−1
448	IFNA1	1	down	−1
449	IFNA10	1	down	−1
450	IFNA13	1	down	−1
451	IFNA14	1	down	−1
452	IFNA16	1	down	−1
453	IFNA17	1	down	−1
454	IFNA2	1	down	−1
455	IFNA21	1	down	−1
456	IFNA4	1	down	−1
457	IFNA5	1	down	−1
458	IFNA6	1	down	−1
459	IFNA7	1	down	−1
460	IFNA8	1	down	−1
461	IFNAR1	0.604738	up	1.427341
462	IFNAR2	0.639698	up	1.276351
463	IFNB1	1	down	−1
464	IP6K2	0.074849	up	2.206121
465	IRF1	0.385881	up	15.60537
466	IRF2	0.275745	up	1.926423
467	IRF3	0.340062	up	27.52587
468	IRF4	1	down	−1
469	IRF5	1	down	−1
470	IRF6	1	down	−1
471	IRF7	0.205625	up	48.22053
472	IRF8	1	down	−1
473	IRF9	0.373579	down	−17.3882
474	ISG15	0.736064	up	1.159735
475	ISG20	1	down	−1
476	JAK1	0.412642	up	2.032627
477	MX1	0.373579	down	−11.2784
478	MX2	0.904395	up	1.57494
479	OAS1	0.373579	up	26.93757
480	OAS2	0.497302	down	−1.80768
481	OAS3	0.928764	up	1.042124
482	OASL	1	down	−1
483	PSMB8	0.953498	down	−1.01913
484	PTPN1	0.421216	up	2.896049
485	PTPN11	0.700305	up	1.02794
486	PTPN6	0.466161	up	1.838392

487	RNASEL	0.482366	down	−1.21913
488	RSAD2	0.373579	down	−8.84184
489	SAMHD1	1	down	−1
490	SOCS1	1	down	−1
491	SOCS3	0.373579	up	9.598569
492	STAT1	0.380206	up	1.448652
493	STAT2	0.421894	up	1.463777
494	TYK2	0.190994	down	−1.89623
495	USP18	0.469187	up	1.571843
496	XAF1	1	down	−1
		**NFκB**		
497	ABHD8	1	down	−1
498	ACTN1	0.68631	down	−1.1372
499	ACTN3	1	down	−1
500	ADGRB2	0.84726	up	1.88967
501	AGPAT1	0.60671	down	−1.455
502	ALG6	0.35601	up	14.8678
503	AP1S2	0.62617	up	1.82384
504	ARHGAP44	1	down	−1
505	ARHGAP5	0.09871	up	1.20635
506	ARHGAP8	1	down	−1
507	ARPC2	0.82457	down	−1.0802
508	ASCL3	1	down	−1
509	ASH1L	0.06049	down	−2.8156
510	ATOH1	1	down	−1
511	ATP1B1	0.98293	up	1.07859
512	BCKDK	0.66906	up	1.1586
513	BCL3	0.91929	up	1.46704
514	BCL6B	0.37358	up	8.81102
515	BDNF	0.37358	down	−8.3694
516	BFSP1	1	down	−1
517	BIRC3	0.9337	down	−1.0856
518	BMF	1	down	−1
519	BMP2K	0.24495	up	1.29685
520	BNC2	1	down	−1
521	C1QL1	1	down	−1
522	CD83	0.93904	down	−1.0643
523	CDC14A	0.91138	down	−1.4913
524	CDK6	0.9421	down	−1.012
525	CFAP69	1	down	−1
526	CHD4	0.32021	up	1.20287
527	CHD6	0.21416	up	1.78502
528	CLCN1	1	down	−1
529	COL11A2	0.88758	up	1.51958
530	COL16A1	1	down	−1
531	CPD	0.50348	up	1.28595
532	CREB1	0.38513	up	1.71716
533	CTAGE4	1	down	−1
534	CTGF	1	down	−1
535	CXCL10	1	down	−1
536	CXCL11	1	down	−1
537	CXCL16	0.93352	up	1.34627
538	CXCL2	1	down	−1
539	CXCL5	1	down	−1
540	CXCL6	1	down	−1
541	CXCL9	1	down	−1
542	CXCR5	1	down	−1
543	CYLD	0.66275	down	−1.3515
544	DAP3	0.10424	down	−2.3797
545	DCLK1	1	down	−1
546	DDR1	0.99666	up	1.0031
547	EBI3	1	down	−1
548	EDN2	1	down	−1
549	EGF	1	down	−1
550	EHF	1	down	−1
551	EIF5A	0.0308	down	−2.8691
552	ENO3	0.37358	up	12.326
553	ERN1	0.41865	up	1.56566
554	ETV6	0.09165	up	1.71061
555	FBXL12	0.28571	up	1.56443
556	FGF1	1	down	−1
557	FGF17	1	down	−1
558	FLRT1	1	down	−1

559	FOXJ2	0.70552	down	−1.1583
560	FOXS1	1	down	−1
561	FTHL17	1	down	−1
562	FUT7	0.98263	up	1.07522
563	GABRB1	1	down	−1
564	GADD45B	0.35651	up	15.4135
565	GATA4	1	down	−1
566	GEN1	0.65117	up	1.29679
567	GNAO1	0.37358	down	−5.9641
568	GNB1	0.20052	up	1.18781
569	GNG4	1	down	−1
570	GPHN	0.16979	up	2.38881
571	GREM1	1	down	−1
572	GRIN2D	1	down	−1
573	GRK5	0.47382	up	7.85783
574	HCFC1	0.29852	up	1.31879
575	HCST	0.99556	up	1.0275
576	HIVEP1	0.08069	up	1.71572
577	HOXA11	0.92972	down	−1.3656
578	HOXB9	1	down	−1
579	HSD11B2	1	down	−1
580	HSD3B7	1	down	−1
581	ICAM1	0.37358	up	8.77461
582	IER5	0.73809	down	−1.1812
583	IFNB1	1	down	−1
584	IGDCC3	1	down	−1
585	IL13	1	down	−1
586	IL1A	1	down	−1
587	IL1RAPL1	1	down	−1
588	IL1RN	1	down	−1
589	IL23A	2.04E-07	down	−276.95
590	IL27	1	down	−1
591	IL27RA	0.30762	up	27.1906
592	IL2RA	1	down	−1
593	IL4I1	1	down	−1
594	INO80D	0.66617	up	1.35449
595	IRF1	0.38588	up	15.6054
596	ITGB4	1	down	−1
597	JAK3	0.16216	up	2.53706
598	JARID2	0.50047	up	1.17656
599	KANSL1L	0.37358	down	−13.248
600	KCNH3	0.37358	down	−8.1217
601	KCNN3	1	down	−1
602	KCNS3	1	down	−1
603	KLK9	1	down	−1
604	KRTAP13-1	1	down	−1
605	LAMA1	1	down	−1
606	LIG1	0.04207	up	2.20869
607	LRCH1	0.579	up	1.60042
608	LTA	1	down	−1
609	LTB	1	down	−1
610	MADCAM1	1	down	−1
611	MAML2	1	down	−1
612	MAP3K8	0.37358	down	−6.6771
613	MAP4K2	0.01066	down	−2.4515
614	MED1	0.63561	up	1.19448
615	MMP9	1	down	−1
616	MOB3C	7.34E-07	down	−84.746
617	MSC	1	down	−1
618	MSN	0.04512	up	1.96984
619	MSX1	1	down	−1
620	NFAT5	0.0983	down	−1.6318
621	NFKB2	1.18E-04	down	−253.27
622	NFKBIB	0.60382	down	−1.2591
623	NFKBID	0.00159	down	−175.53
624	NLK	0.27105	up	17.2459
625	NOL4	1	down	−1
626	NR2F2	1	down	−1
627	NTN1	1	down	−1
628	OPCML	1	down	−1
629	P2RY10	1	down	−1
630	PAN2	0.05787	up	2.28619
631	PCBP4	0.41539	up	1.90928

632	PCSK2	1	down	−1
633	PFN1	0.65884	down	−1.0696
634	PLAU	1	down	−1
635	PRDM12	1	down	−1
636	PRX	0.37358	down	−7.0651
637	PTMS	0.69111	down	−1.3869
638	RALGDS	0.90585	down	−1.0967
639	RAP2C	0.14359	down	−1.2295
640	RBMS1	0.13269	up	1.70083
641	RELB	0.94154	down	−1.3132
642	RIMS2	0.98946	up	1.03369
643	RIN2	1	down	−1
644	RND1	0.00194	up	241.729
645	RPS6KA4	0.4893	up	1.17319
646	RRAS	1	down	−1
647	SALL1	1	down	−1
648	SAMSN1	0.15407	down	−2.1742
649	SDC4	1	down	−1
650	SEC14L2	0.48338	down	−7.0382
651	SESN2	0.32328	up	16.2986
652	SHOX2	0.37358	up	10.0979
653	SIN3A	0.46729	up	1.34898
654	SLAMF8	0.37358	up	17.4482
655	SLC11A2	0.16926	down	−1.9723
656	SLC12A2	0.83201	up	1.27654
657	SLC6A12	1	down	−1
658	SMOC1	1	down	−1
659	SMPD3	0.39987	up	2.01754
660	SOBP	0.99171	up	1.02976
661	SOX10	1	down	−1
662	SOX5	1	down	−1
663	SP6	1	down	−1
664	SPTB	1	down	−1
665	STAT6	0.69937	down	−1.2906
666	STC2	1	down	−1
667	SUN2	0.37071	up	1.6998
668	TAL1	0.85741	up	1.06796
669	TAZ	0.90592	down	−1.1136
670	TCEA2	0.37358	up	13.4054
671	TLX1	1	down	−1
672	TNF	1.10E-05	up	170.291
673	TNFRSF1B	0.10108	down	−2.5365
674	TNFRSF9	1	down	−1
675	TNFSF15	1	down	−1
676	TNFSF18	1	down	−1
677	TNIP1	0.27302	down	−1.4924
678	TP53	0.04771	up	1.80401
679	TP63	1	down	−1
680	TRAF4	0.69005	up	1.35819
681	TRIB2	0.91094	down	−1.0969
682	TRIM47	0.37358	down	−21.308
683	TSLP	0.92669	up	1.52376
684	TUBGCP4	0.43011	down	−13.158
685	UACA	1	down	−1
686	UBD	1	down	−1
687	UBE2D3	0.47358	down	−1.1466
688	UBE2H	0.25753	up	1.78271
689	UBE2I	0.35498	up	1.33321
690	UBE4B	0.30625	down	−1.4813
691	VCAM1	1	down	−1
692	VSX2	1	down	−1
693	WNT10A	1	down	−1
694	WNT10B	1	down	−1
695	ZBTB11	0.6547	up	1.30638
696	ZBTB5	0.37505	up	11.3093
697	ZBTB9	0.28811	up	18.3014
698	ZFP36L2	0.17509	down	−1.3444
699	ZMYND15	1	down	−1
700	ZNF232	0.95499	down	−1.2557
701	ZNF384	0.93961	down	−1.047
702	ZNF821	1	down	−1
